# Genomic deregulation of PRMT5 supports growth and stress tolerance in chronic lymphocytic leukemia

**DOI:** 10.1038/s41598-020-66224-1

**Published:** 2020-06-17

**Authors:** Ann-Kathrin Schnormeier, Claudia Pommerenke, Maren Kaufmann, Hans G. Drexler, Max Koeppel

**Affiliations:** 1 Leibniz-Institute DSMZ-German Collection of Microorganisms and Cell Cultures, Department of Human and Animal Cell Lines, Braunschweig, Germany; 2 Present Address: Institute for Cell Biology (Tumor Research), University of Duisburg-Essen, Medical School, Duisburg, Germany

**Keywords:** Haematological cancer, Cancer genomics

## Abstract

Patients suffering from chronic lymphocytic leukemia (CLL) display highly diverse clinical courses ranging from indolent cases to aggressive disease, with genetic and epigenetic features resembling this diversity. Here, we developed a comprehensive approach combining a variety of molecular and clinical data to pinpoint translocation events disrupting long-range chromatin interactions and causing cancer-relevant transcriptional deregulation. Thereby, we discovered a B cell specific cis-regulatory element restricting the expression of genes in the associated locus, including PRMT5 and DAD1, two factors with oncogenic potential. Experimental PRMT5 inhibition identified transcriptional programs similar to those in patients with differences in PRMT5 abundance, especially MYC-driven and stress response pathways. In turn, such inhibition impairs factors involved in DNA repair, sensitizing cells for apoptosis. Moreover, we show that artificial deletion of the regulatory element from its endogenous context resulted in upregulation of corresponding genes, including PRMT5. Furthermore, such disruption renders PRMT5 transcription vulnerable to additional stimuli and subsequently alters the expression of downstream PRMT5 targets. These studies provide a mechanism of PRMT5 deregulation in CLL and the molecular dependencies identified might have therapeutic implementations.

## Introduction

Among hematopoietic malignancies chronic lymphocytic leukemia (CLL), a disorder of mature B cells, represents one of the most common forms of neoplasms with an incidence of 4–5 cases in 100,000 per year according to the National Cancer Institute of USA (SEER: https://seer.cancer.gov/statfacts/html/clyl.html). Stratification of patients into clinical risk groups is mainly based on the mutational status of the immunoglobulin variable regions, with IGHV-unmutated CLLs displaying a worse prognosis than IGHV-mutated cases^1^, but also changes in transcriptional networks have successfully been used to assign patients to such groups^2^. These different approaches together with recent therapeutic improvements like the application of monoclonal antibodies and kinase inhibitors improved prognosis, yet the overall outcome remains diverse and CLL mostly an incurable disease^3–5^.

Recently, extensive patient centered research succeeded in characterizing the molecular landscape of CLL, with only few individual genes mutated at rates over 10% and highly recurrent events occurring only as some copy number alterations^6,7^, defining the genomic features of CLL as heterogeneous as its clinical outcome. In parallel, the comprehensive dissection of the epigenetic landscape defined further subgroups of CLL based on the differentiation status of leukemic cells and chromatin accessibility of individual transcription factors, highlighting aberrant reprogramming events^8,9^.

Among epigenetic modifiers with a pivotal role during hematopoietic development, the type II arginine methyl-transferase PRMT5 maintains the balance between quiescent, non-differentiated cells and proliferation of lineage committed ones^10,11^. Moreover, during the formation of germinal centers, the abundance of PRMT5 increases and it interacts with and supports the gene-repressive function of BCL6^12,13^. While aberrant expression of PRMT5 favors transformation and tumor growth in a range of hematologic as well as solid and soft tissue neoplasms^14–17^, the events causing PRMT5 deregulation during cellular transformation remain largely uncharacterized.

Here, we identified an event of PRMT5 deregulation in CLL by performing thorough analyses of genomic, transcriptomic, functional, and clinical data. Our analysis of PRMT5 function in CLL also unravels a crosstalk with crucial growth regulatory factors, like the MYC network taking place *in vitro* and in CLL patients. It further indicates that PRMT5 maintains the abundance of factors involved in the DNA-repair, resulting in increasing apoptosis if simultaneously inhibited. Furthermore, we use CRISPR/cas9 genomic engineering to mimic the disruption of the regulatory loop and find that loss of the upstream region causes an increase in PRMT5 expression and additional imbalances in the transcriptional regulation of the associated locus. Subsequently, we show that regulation of target genes and the observed phenotype are opposite to the ones seen upon PRMT5 inhibition and fit the observations made in CLL donors with high PRMT5.

## Results

### Identification of candidate genes with translocation caused deregulation

**Figure 1:**
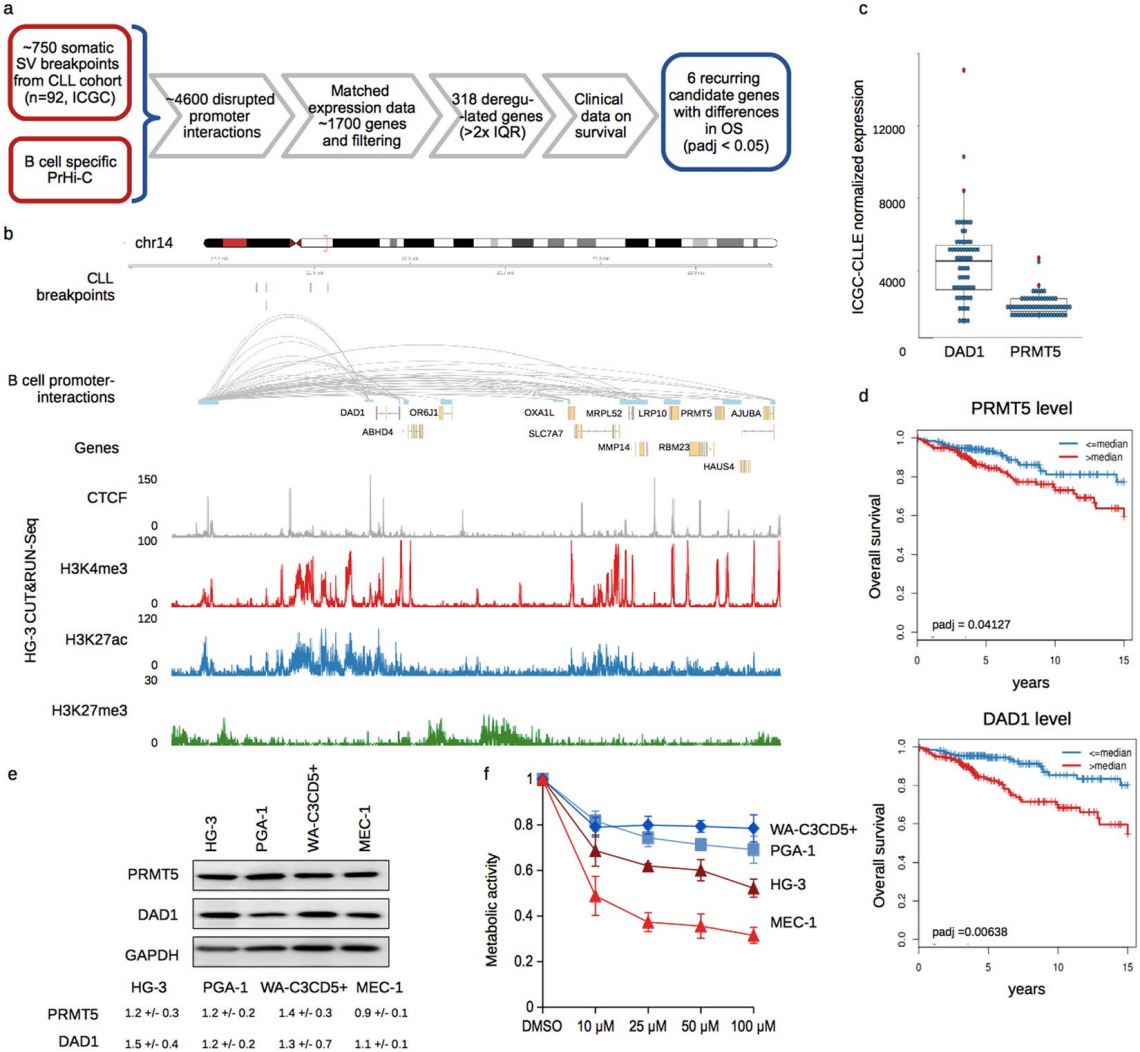
PRMT5 and DAD1 as candidate cancer-genes deregulated through SV in CLL. (**a**) Workflow to identify genomic breakage-caused aberrant cancer-gene expression (SV: structural variations; PrHi-C: Promoter-HiC; IQR: Interquartile range; OS: Overall survival). (**b**) Schematic representation of the locus on chr14 harboring DAD1 and PRMT5 with a disrupted cis-regulatory region and its epigenetic make-up in HG-3 cells. Below the genomic coordinates, genomic breakpoints from donors of the ICGC-CLLE cohort are depicted, followed by promoter-interactions (PrHi-C) of the indicated genes derived from total B cells within the IHEC (interactions in grey, anchor-regions in lightblue, heights correspond to published score). CUT&RUN tracks of CTCF (grey), H3K4me3 (red), H3K27ac (blue) and H3K27me3 (green) derived from HG-3 cells are shown below the gene annotation track. (**c**) Expression of DAD1 and PRMT5 in CLL donors of the ICGC-CLLE cohort for which breakpoint- and transcriptional data were available (donors with a breakpoint upstream of DAD1 in red, other donors in blue). (**d**) Kaplan-Meier plots of overall survival for donors from the ICGC-CLLE^7^ and Herold *et al*.^2^, which were grouped based on their expression of PRMT5 or DAD1 (padj: Benjamini-Hochberg corrected p-value). (**e**) Protein abundance of PRMT5 and DAD1 in cell lines derived from CLL. GAPDH served as loading control, below the blot median protein abundance relative to control from three biological replicates including standard deviation is indicated. (**f**) Inhibition of metabolic activity upon PRMT5 inhibition. CLL derived cell lines were treated for 96 h with increasing concentrations of the PRMT5 inhibitor EPZ015666 (EPZ), followed by MTT assay (Error bar represents SD of three independent experiments).

To evaluate effects of structural variations on gene expression and tumor progression, we applied an integrative approach to find factors with deregulated expression in cancer patients which might contribute to the disease (Fig. 1a). Specifically, we extracted ~750 chromosomal breakpoints from 92 donors of the ICGC cohort on chronic lymphocytic leukemia (ICGC-CLLE)^7^. To link breakpoints with individual genes likely to suffer from disrupted transcriptional regulation, we included a B cell specific set of promoter-interactions (PrHi-C)^18^. This allowed us to distinguish genes in close proximity to breakpoints that might be affected, from those with unaffected regulatory interactions, on which aberrations therefore most likely do not have an effect. This identified ~4,600 disrupted interactions affecting ~1,700 unique genes, 318 of which exhibited alterations in their expression larger than two times the interquartile range (IQR) for that gene across all patients. Out of these 318 genes, we found that 47 genes were recurrently deregulated by at least one IQR in two or more patients.

Next, we assessed whether the expression of any of these 47 genes had an influence on disease outcome. To enhance statistical power, we included another cohort of patients^2^ with publicly available clinical and expression data and divided patients into high or low expressing groups separated by the median expression value for each of the 47 candidate factors. Survival analysis (Log-rank test) indicated 6 factors for which statistical differences in overall survival (OS) could be observed (Benjamini-Hochberg corrected p-value < 5 * 10^−2^; Table 1). We put a focus on potentially deregulated gene clusters, where promoter-interactions of several genes to the same regulatory site were interrupted and thereby identified a locus on chr14 (Fig. 1b, upper and middle panel).

**Table 1:** Candidate genes with potential genomic deregulation affecting overall survival.

Identified gene	p-Value OS	padj. OS	Genomic coordinates	Disrupted PIR-anchor	Number of donors with corresponding event
TFAP2A-AS1*	0.00032	0.01472	chr6:10409340-10416446	chr6:99589388-99626140	3
DLX2	0.00288	0.04127	chr2:172099439-172102900	chr2:63042965-63063823	2
DAD1	0.00016	0.00638	chr14:22564905-22589269	chr14:22370281-22410008	2
ZNF142	0.00521	0.04164	chr2:218637916-218659655	chr2:128075688-128101753	2
ELP4	0.00354	0.04127	chr11:31509700-31790328	multiple	3
PRMT5	0.00413	0.04127	chr14:22920511-22929585	chr14:22370281-22410008	2

Genes identified by our computational approach with a genomic breakpoint disrupting interactions between promoters and their interacting regions (PIR) and with a corresponding expressional change > 2x IQR across the patients with genomic and transcriptomic data available. For each of the identified candidates high versus low expressing CLL donors from Puente *et al*.^7^ and Herold *et al*.^2^ were analyzed by Log-rank test for differences in overall survival (OS; padj: Benjamini-Hochberg corrected p-value). *Due to different methodology, TFAP2A-AS1 was covered only by Puente *et al*.^7^ but not by Herold *et al*.

The genomic breakpoints disrupting promoter-interactions in the respective donors (2 out of 92) were associated with the induction of several genes, like DAD1 and PRMT5 above our IQR threshold (Fig. 1c, and Supplemental Fig. S1a). These genes were of particular interest as their expression correlated with differences in overall survival in the combined cohorts of CLL patients (Fig. 1d). Aiming to evaluate additional potential risk factors possibly associated with high PRMT5 expression, we compared the occurrence of known genomic aberrations like TP53 mutations or frequently occurring chromosomal aberrations like 11q deletions (Supplemental Table S1). This showed a slightly higher number of IGHV unmutated donors in the PRMT5 high group, and also an increased number of TP53 mutations, both events considered to confer poor prognosis. We also noted that while the donors with disrupted regulatory interactions were those with the highest PRMT5 expression, we found in total nine cases in which PRMT5 expression exceeded an increase >1 IQR, pointing likely to different mechanisms of PRMT5 deregulation. To obtain more insight into the locus, we assessed the epigenetic state of the respective locus in the CLL cell line HG-3 by a nuclease-tethering strategy (CUT&RUN)^19^. The upstream regulatory region was demarcated by CTCF and active histone marks, while it was devoid of H3K27me3 (Fig. 1b). While most of the genes across the locus showed active regulatory promoter-marks together with CTCF binding-sites, H3K27me3 was mainly present between genes. To further expand our examination of this locus, we analyzed several reference epigenomes from the BLUEPRINT encompassing various normal B cell subtypes as well as CLL samples^8^ (Supplemental Fig. S1b) and found the epigenomic landscape highly similar across all cells of B cell origin. Immediately upstream of DAD1 a high accumulation of H3K27ac and H3K4me3 indicated a so-called super enhancer (Supplemental Fig. S1c), and it showed slightly more activity in CLL samples compared to non-malignant B cells. The anchor region ~200 kb upstream of DAD1 harbored a strong enhancer in all analyzed B cell subtypes, resembled also by the presence of a sharp H3K27ac signal, which was not present in T cells, in which promoter-interactions of the respective genes did not include this upstream region of interest. We examined the abundance of PRMT5 and DAD1 in a range of cell lines derived from CLL and the related mantle cell lymphoma (MCL)^20,21^ and found them to be expressed at detectable, yet varying levels (Fig. 1e and Supplemental Fig. S2a,b). In addition, we used the same BLUEPRINT reference data to examine the expression of these two genes in the same group of B cell subsets and CLL samples (Supplemental Fig. S2c). We found PRMT5 to be higher expressed in CLL samples than in memory B cells, but lower than in germinal center B cells, while DAD1 showed overall higher expression but also higher variability in CLL samples. Assessing the physiological relevance of PRMT5 in CLL, we treated different CLL derived cell lines for 96 h with increasing concentrations of the PRMT5-specific inhibitor EPZ015666 and followed their metabolic activity. MEC-1 and HG-3 cells displayed higher sensitivity, already at low concentrations, while PGA-1 and WA-C3CD5 + cells were only moderately affected (Fig. 1f). With this approach we could not only identify potentially deregulated genes, having an effect on the clinical prognosis of CLL, but also showed that the inhibition of one of them, PRMT5, had a growth reducing effect in CLL derived cell lines.

### PRMT5 maintains growth supporting transcriptional states, including MYC-driven programs *in vitro* and *in vivo*

**Figure 2:**
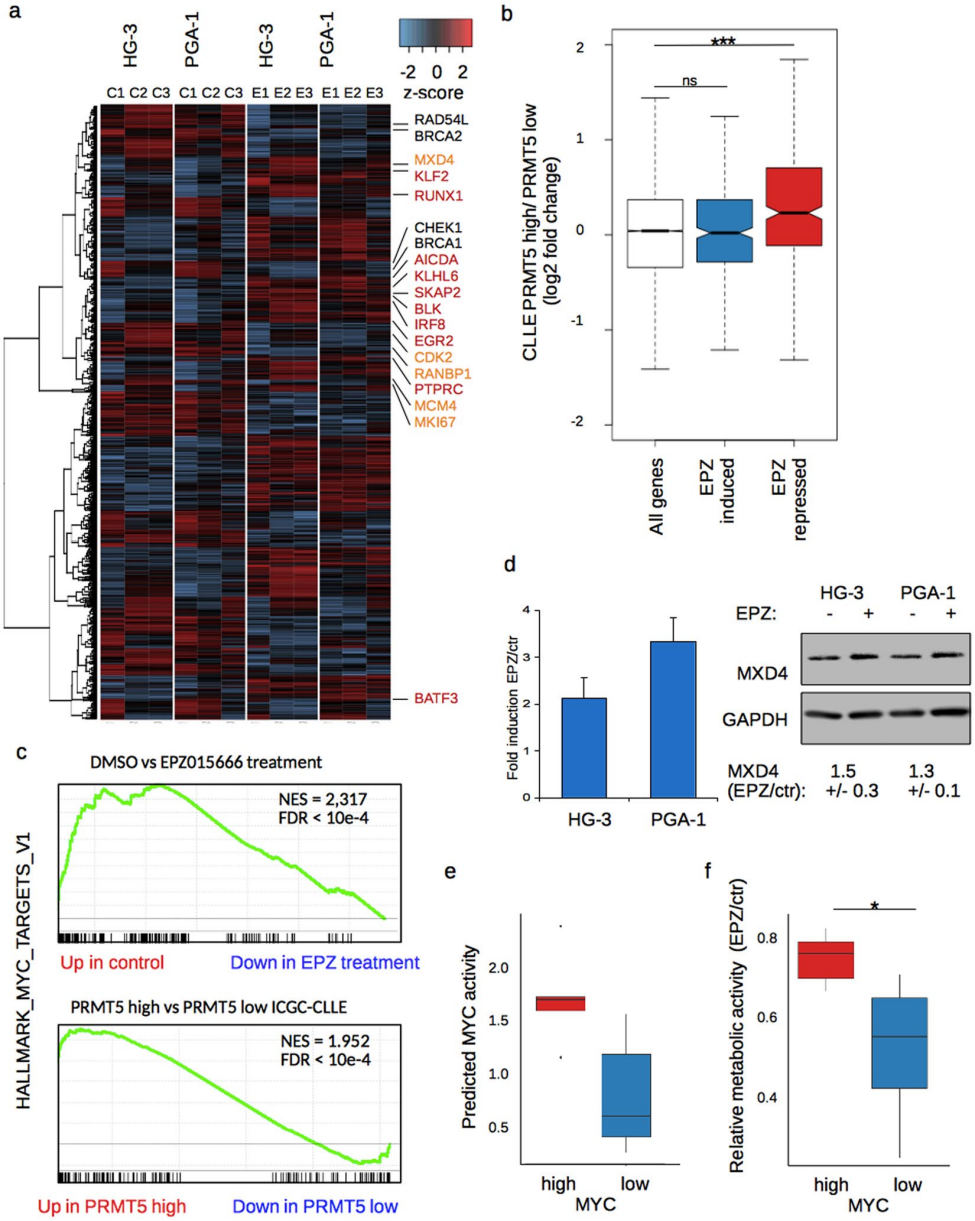
PRMT5 supports growth promoting pathways in vitro and *in vivo*. (**a**) Heatmap showing up- and downregulated genes of three replicates of RNA-seq per cell line, calculated by DESeq. 2 (padj < 0.05, logFC > |0.6|). C1-3 represent untreated control replicates, E1-3 those treated with 10 µM EPZ015666 for 96 h. Highlighted are genes involved in B cell and lymphocyte specific pathways (red), cell cycle progression (orange) and checkpoint control (black). b) Correlation between transcriptional changes of EPZ015666 treated cells and donors from the CLLE cohort. Boxplot depicts log2 fold change in expression between PRMT5 high versus PRMT5 low donors, for all genes (white), genes induced (blue) or repressed (red) upon EPZ015666-treatment, respectively. Statistical testing by Mann-Whitney-U test (***p < 10^-12^). (**c**) GSEA for control versus EPZ015666 treated HG-3 and PGA-1 cells (upper panel) and for PRMT5 high versus PRMT5 low CLL donors (lower panel) shows a downregulation of hallmark MYC target V1 gene set. (**d**) Upregulation of MXD4 upon treatment of HG-3 and PGA-1 cells with EPZ015666, shown at the mRNA-level (left) and protein (right), GAPDH served as loading control. Below the blot the median fold change of treated over control of protein abundance was calculated including the SD from three independent experiments. (**e**) Boxplot shows the transcriptional activity of MYC in the MYC high and MYC low cell line groups, estimated via DoRothEA v2. (**f**) Metabolic activity upon PRMT5 inhibition in the two cell line groups with high or low MYC activity as determined by MTT (statistical testing by student’s paired T-test; *p < 0.01).

To elucidate the underlying molecular programs, we performed RNA-seq analysis upon PRMT5 inhibition. In order to avoid a strong and potential secondary apoptotic response, we selected the two moderately affected cell lines PGA-1 and HG-3 and detected 1166 genes changing their expression (padj < 0.05; log2 > |0.6|) in the combined dataset (Fig. 2a). Approximately half of them were up- or downregulated respectively, notably with several of the induced genes related to lymphocyte function and repressed ones involved in proliferation and cell cycle control, of which several clustered close to each other regarding their expression level. We also performed principal component analysis (PCA) to assess their global changes and found that the first component, explaining almost 50% of variance between samples, resulted from inhibitor treatment (Supplemental Fig. S3a). To ensure that our observation would be reflected in clinical settings, we included expression data from the ICGC-CLLE cohort in our analysis and ranked patients based on their PRMT5 expression level. After performing differential expression analysis of the 10% CLLE donors with the highest PRMT5 level (n = 30) versus the 10% showing the lowest expression (n = 30), we compared the output with the differential expression data from our PRMT5 inhibitor experiments. Remarkably, we found genes that become repressed upon treatment with EPZ015666 were overall significantly higher expressed in PRMT5-high CLLE donors (Mann-Whitney-U test; p < 10^−12^) (Fig. 2B and Table S2), indicating that experimentally derived PRMT5 effects are reflected *in vivo*.

Using gene set enrichment analysis (GSEA) with the same expression data, we found significant transcriptional reduction of the HALLMARK gene sets for MYC- and E2F-target genes as well as those responsible for the G2/M checkpoint, similarly in our inhibitor data and the CLLE cohort (Fig. 2c and Supplemental Fig. S3b). Despite the fact that we did not detect any changes in the expression levels of MYC itself, the Upstream Regulator Analysis of the Ingenuity Pathway Analysis- (IPA-) software indicated a role for MYC in the transcriptional responses upon PRMT5-inhibition (Supplemental Fig. S3c). Hence, we screened for factors known to influence the activity of MYC and found the MYC-antagonist MXD4 to be among our expression changing genes. Additional validation confirmed an increase of MXD4 mRNA and showed a 30–50% increase in the amount of protein upon PRMT5 inhibition in HG-3 and PGA-1 cells (Fig. 2d), which could contribute to the reduced MYC activity we observed. Further, we speculated whether high activity of MYC could influence the physiological consequences of PRMT5 inhibition. Besides CLL and MCL cell lines, we included three additional cell lines with activating MYC-fusions (Burkitt lymphoma cell lines RAMOS, DAUDI, VAL). First we grouped the cell lines according to their mRNA-levels of MYC, based on previously generated transcriptomic data^22^ (Supplemental Fig. S3d) and divided the cell lines into a MYC high (RAMOS, JEKO-1, MINO, REC-1, DAUDI, WA-C3CD5+) and a MYC low (VAL, PGA-1, MEC-1, HG-3, JVM-2, GRANTA-519) group. In a next step, we assessed the activity state of MYC in those two groups by using previously generated predictions of transcription factor activity from the DoRothEA algorithm^23^, and find high levels of MYC-mRNA to correlate well with high levels of transcription factor activity (Fig. 2e). We next compared the effects of PRMT5 inhibition on the metabolic activity in the two groups. Strikingly, we find that high levels of MYC confer reduced sensitivity to PRMT5 inhibition (Fig. 2f), supporting an activating crosstalk between the two factors. In summary, our RNA-seq data resembles the patient-derived transcriptional profiles and molecular pathways appear similarly active, both *in vitro* and *in vivo*.

### PRMT5 inhibition blocks DNA repair pathways in CLL cell lines

**Figure 3:**
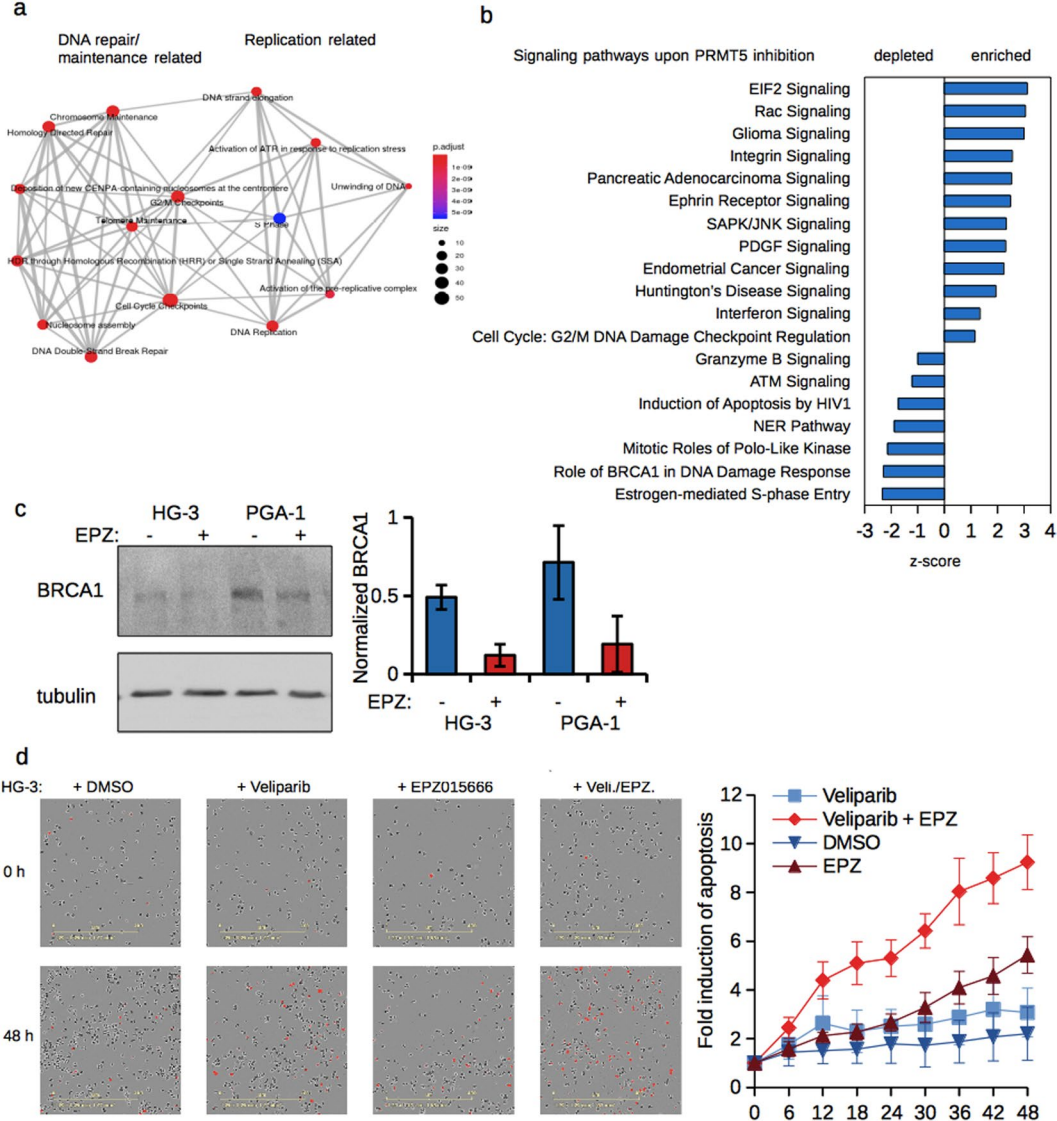
PRMT5 inhibition impairs stress response pathways and sensitizes cells to PARP inhibitors. (**a**) Reactome pathway analysis of the expression changing genes common to both cell lines. Emapplot showing top 15 categories of enrichment, with dot-size representing number of genes in the category and color representing adjusted p-value. (**b**) Ingenuity Pathway Analysis (IPA) depicts enriched or depleted pathways in differential expression data between control and EPZ015666-treated cells (pval < 0.05 and z-score > |1|). (**c**) Western blot for BRCA1 in control and EPZ015666-treated cells (left panel). Tubulin served as loading control. Quantification of BCRA1 abundance in control and EPZ015666-treated cells relative to tubulin with error bars representing SD from three biological replicates (right panel). (**d**) IncuCyte Caspase-3/7 assay depicts representative pictures showing the induction of apoptosis in HG-3 cells treated as indicated after 0 and 48 h (left panel). Right panel shows summary graphs of the induction of apoptosis over time and with the indicated treatments (error bars represent SD from three biological replicates).

To further categorize regulated genes within the combined dataset of HG-3 and PGA-1 cells, we employed Reactome Pathway analysis and found expression changing genes mainly related to processes of genomic replication as well as DNA-repair/-maintenance and checkpoint control (Fig. 3a). To further extend our characterization of PRMT5 regulated pathways, we used Ingenuity Pathway Analysis to identify enriched or depleted signaling nodes and cascades within the regulated genes (Fig. 3b). While we found several cancer signaling pathways enriched in PRMT5 inhibited cells, we also observed a depletion of pathways involved in various DNA repair pathways, such as BRCA1, nucleotide excision repair (NER) and ATM signaling. Additional analysis, individually in HG-3 and PGA-1 cells, on groups of either up- or downregulated genes, linked genes with reduced expression to categories of DNA-replication and chromatin organization (Supplemental Fig. S4a). Upregulated genes however showed some enrichment of translational related processes only in HG-3 cells (Supplemental Fig. S4b).

As checkpoint and DNA repair pathways were predicted to have reduced activity, we focussed on BRCA1, as a central regulator of repair processes. Inhibition of PRMT5 caused both, reduced levels of BRCA1 mRNA as well as protein (Figs. 2a and 3c). As BRCA1 deficiency confers a particular sensitivity to PARP inhibition, we tested whether such inhibitors would similarly exceed stress tolerance in PRMT5 inhibited cells. Hence, we assessed the induction of apoptosis by PRMT5 inhibition individually or combined with the PARP inhibitor Veliparib by live cell imaging in HG-3 cells as they showed a particular reduction in Homology Directed and DNA double-strand break repair (Supplemental Fig. S4a). Following the activation of a dye-linked caspase 3/7 substrate as a surrogate for the induction of apoptosis, we found a strong increase in cell death upon combination treatment over the 48 h monitoring period, linking molecular reduction of DNA-repair pathways with a lowered apoptotic threshold (Fig. 3d). Thereby we could show that particular pathways, affected by PRMT5 inhibition, can be exploited to trigger cell death.

### Deregulation of PRMT5 and DAD1 by loss of regulatory region

**Figure 4:**
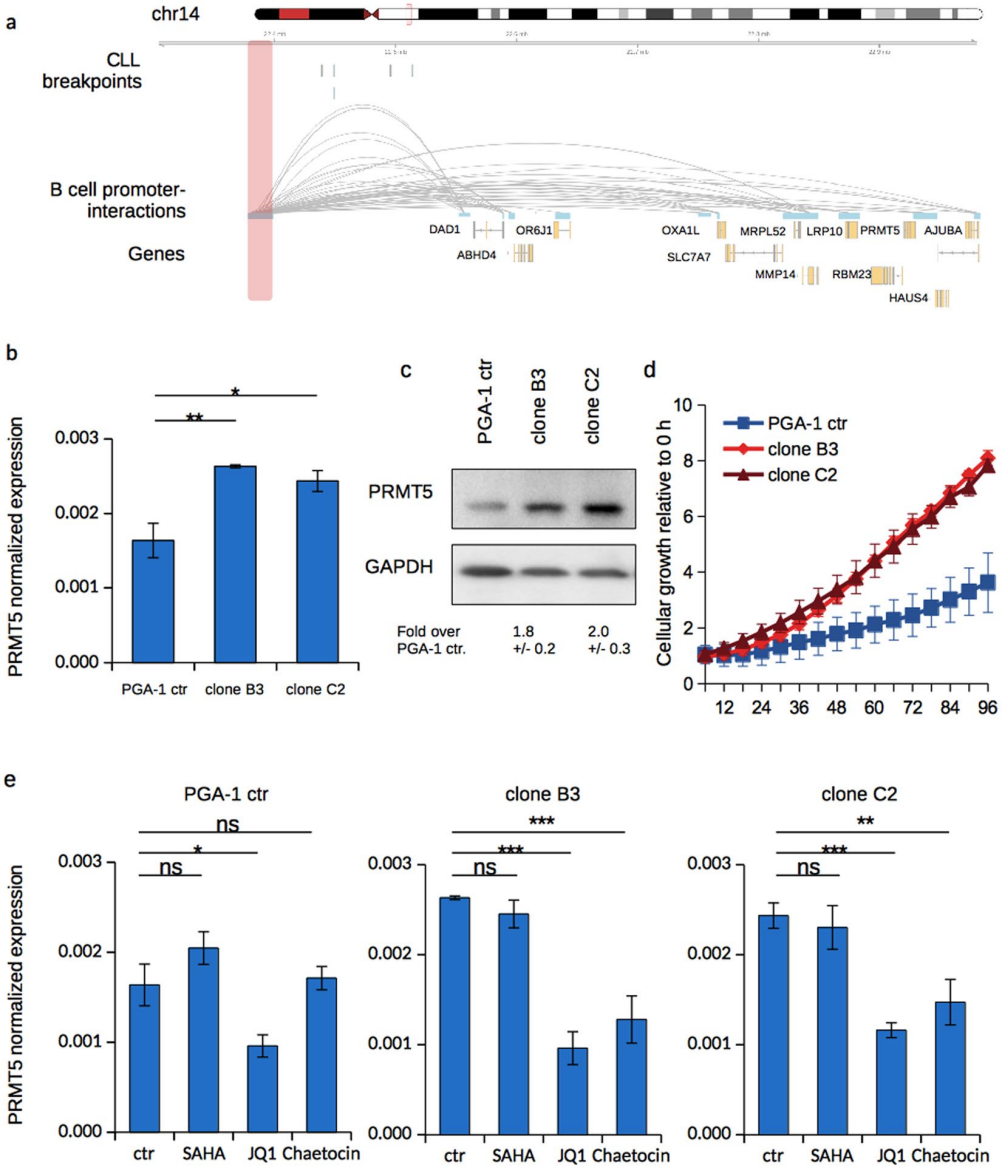
Deregulation of PRMT5 by loss of its regulatory region. (**a**) Below the genomic coordinates, genomic breakpoints from donors of the ICGC-CLLE cohort are depicted, followed by promoter-interactions (PrHi-C) of the indicated genes. Red box indicates regulatory region excised with CRISPR/cas9. (**b**) Normalized PRMT5 mRNA expression analyzed by RT-qPCR of PGA-1 clones with a CRISPR/cas9 engineered deletion of the upstream regulatory region and compared to PGA-1 control for which cells were transduced with a construct lacking the guideRNA (error bars represent SD from three biological replicates). (**c**) Western blot for PRMT5 in the same PGA-1 clones lacking the upstream regulatory region. GAPDH served as loading control and was used to normalize PRMT5 level. Below the blot levels of normalized PRMT5 compared to the control PGA-1 clone are shown (SD derived from three biological replicates). (**d**) Growth rates of the PGA-1 clones and control cells measured by IncuCyte live cell imaging for a total of 96 h (confluence was measured; error bars resemble SD from three biological experiments, each performed in triplicate). (**e**) Cells from the PGA-1 control or the respective clones were treated with 1 µM SAHA or 5 µM JQ1 for 48 h or 50 nM Chaetocin for 24 h prior to the analysis of PRMT5 mRNA via RT-qPCR. Shown is normalized expression (error bars resemble SD from three biological replicates and statistical testing by student’s paired T-test; *p < 0.05; **p < 0.005; ***p < 0.001).

Finding cases of aberrant PRMT5 expression linked to the disruption of a corresponding regulatory unit in CLL, we aimed to recapitulate these genomic events to unravel a potential effect of this region on the expression of the corresponding genes. As we found the regulatory landscape in this locus to be highly similar in normal B cells, CLL donors and cell lines (Supplemental Fig. S1b), we assumed a common mechanism of regulation and chose PGA-1 cells as our model system, since they showed lower variance in protein levels compared to HG-3 (Fig. 1e). After deletion of the whole endogenous anchor-region on chr14 by CRISPR/cas9 genomic engineering (Supplemental Fig. S5a,b), we subsequently monitored the expression of PRMT5 in two independent clones. Strikingly, PRMT5 was reproducibly upregulated in both derived clones, compared to transduced control cells lacking the respective guideRNAs (Fig. 4b). Similarly, DAD1 and OXA1L showed increased transcriptional levels (Supplemental Fig. S6a). When assessing the levels of the corresponding proteins we observed an induction of all three factors (Fig. 4c and Supplemental Fig. S6b).

We then examined putative physiological changes in the two clones by following their growth rate via live cell imaging. Interestingly, the derived clones revealed an enhanced growth phenotype compared to control cells (Fig. 4d). While deletion of the upstream regulatory site increased PRMT5 expression, we next asked whether it might have additional regulatory functions, for example during a response of cells to external stimuli. To this end we applied different epigenetic active substances, the HDAC inhibitor SAHA, JQ1, a BET-inhibitor mainly blocking BRD4 activity, and Chaetocin, an inhibitor of histone-lysine methyltransferases, and monitored PRMT5 expression. In the control, non-guideRNA cells, SAHA had a modest effect on PRMT5 expression, yet it did not change the expression in the tested regulatory clones, while JQ1 reduced PRMT5 transcript levels in all cases, presumably due to its inhibitory function towards the DAD1 super-enhancer (Fig. 4e, Supplemental Fig. S1b, c). Interestingly, the inhibition of histone-lysine methyltransferases did not cause any effect in the control cells, while it significantly reduced PRMT5 expression in both clones. Based on these results, we concluded that the upstream regulatory region indeed has an influence on the regulation of PRMT5 and might furthermore serve as a stabilizing element in transcriptional responses to external stimuli.

### Downstream effects of genomic PRMT5 activation

**Figure 5:**
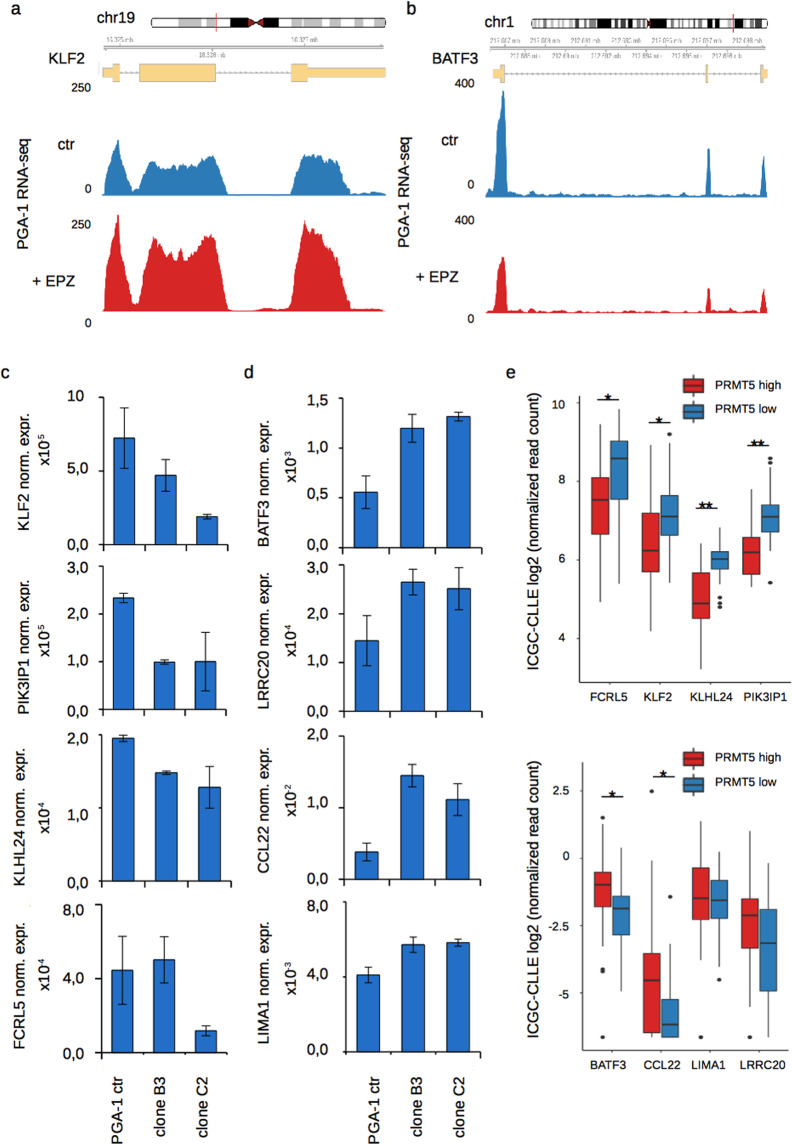
Downstream transcriptional changes after PRMT5 deregulation. (**a**) Below the genomic coordinates and the gene-structure of KLF2, RNA-seq tracks control (blue) and EPZ015666 treated (red) cells are displayed. (**b**) Similar display for BATF3, tracks as displayed in (**a**). (**c,d**) Normalized expression levels assessed by RT-qPCR from PGA-1 clones transduced with a non-guideRNA control or the regulatory deletion clones for the indicated genes previously found activated by PRMT5 inhibition (**c**), or for genes with impaired expression after PRMT5 inhibition (**d**) (error bars represent SD from three biological replicates). (**e,f**) Normalized expression from donors of the CLLE cohort with either PRMT5 high (red) or low (blue) PRMT5 levels for genes induced after PRMT5 inhibition (**e**) or genes with impaired expression upon EPZ015666 treatment (**f**). Statistical testing by Mann-Whitney-U test; *p < 0.01; **p < 10^-6^.

To also assess downstream molecular effects of increased PRMT5 levels, we examined the expression of several genes that we previously detected to be responsive to PRMT5 inhibition. We initially chose two factors involved in lymphocyte specific growth and differentiation control, which we found to be inversely regulated in our RNA-seq data upon treatment of cells with EPZ015666. An example for increased expression upon PRMT5 inhibition is the Kruppel Like Factor 2 KLF2 (Fig. 5a), which interferes with B cell proliferation upon BCR stimulation and has also been reported to trigger apoptosis when overexpressed^24,25^. A factor repressed upon PRMT5 inhibition, is the Basic Leucine Zipper ATF-like Transcription Factor, BATF3 (Fig. 5b), which was shown to be required for proliferation in Hodgkin lymphoma, anaplastic large cell lymphoma as well as adult T cell leukemia/ lymphoma^26,27^. Subsequently, we followed their expression in the derived regulatory clones and included additional genes that we found deregulated after PRMT5 inhibition (Fig. 5c,d). Monitoring the expression of the EPZ015666 induced genes KLF2, PIK3IP1, KLHL24, and FCRL5, we found all of them to become repressed with higher PRMT5 levels in at least one of the tested clones (Fig. 5c). On the other hand, we tested BATF3 and additional genes requiring PRMT5 for their expression, like LRRC20, CCL22, and LIMA1 (Fig. 5d). Remarkably, these genes showed the expected transcriptional changes as well, with overall higher expression in the regulatory clones, compared to control cells.

Finally, we attempted to follow this correlation between PRMT5 and the tested downstream genes in CLL donors with high or low PRMT5 levels. In line with the previous results, we observed significantly lower levels of PRMT5 suppressed genes in PRMT5 high donors (Mann-Whitney-U test *p < 0.01; **p < 10^-6^) (Fig. 5e). Among the genes that are kept active by PRMT5 we found a significant correlation between PRMT5 level and their expression only for BATF3 and CCL22 (Mann-Whitney-U test *p < 0.01), yet expression of the other two tested candidates showed a similar trend (Fig. 5f).

In summary, the downstream molecular effects in cells that have genomically activated levels of PRMT5 resembled at least partly the expression pattern in CLL donors with high PRMT5 expression and inversely reflected what we observed upon PRMT5 inhibition.

## Discussion

Through integrative analysis of multiple kinds of large scale and patient derived data^2,7,27^ we successfully pinpointed cancer-relevant regulons disrupted uniquely in CLL and identified corresponding candidate genes overexpressed in the respective donors that contributed to overall prognosis. By including clinical as well as cell type specific regulatory data, we extended previous approaches, characterizing aberrant expression patterns related to non-coding genomic alterations and involved in tumor progression^28–31^. Among the identified candidates, we focused on the PRMT5 containing locus and a possible mechanism of its deregulation.

While a crucial function of PRMT5 has been described in hematopoietic progenitors and during the germinal center reaction^11–13^, it displays oncogenic functions in various cancers^10,14,15,32,33^. PRMT5 regulating mechanisms involve CDK4-activity^34^; more recently BCR-signaling in germinal center B cells and in cases of diffuse large B cell lymphoma (DLBCL) has been described to trigger increased PRMT5 expression^35^. As the molecular events at the PRMT5 locus and how they deregulate associated expression during cellular transformation remain still elusive, our findings might represent a more common mechanism of PRMT5 deregulation in B cell lymphomas. This might have remained unnoticed so far, due to the lack of whole genome studies in patient cohorts large enough to detect this disruption of the upstream regulatory site. Due to its prominent role in hematopoiesis, PRMT5 might contribute to lineage definition, and as such a gene it might show high regulatory complexity via multiple enhancer interactions^36,37^.

In line with our observations of concordant PRMT5 and DAD1 deregulation, regulatory complexity within a locus can increase further by simultaneous interactions of different genes with the same regulatory site^38,39^. Additionally, the loss of this upstream regulatory region apparently increased the sensitivity of the locus to transcriptional perturbations caused by particular external stimuli, adding another potential layer of transcriptional control. Downstream transcriptional regulation mediated by PRMT5 showed diverging effects with processes of stress response and DNA-replication maintained by PRMT5 and lymphocyte related pathways suppressed. Among the genes activated upon PRMT5 inhibition were PIK3IP1, FCRL5 and KLF2, which became correspondingly repressed with higher PRMT5 levels. In B cells these genes have mainly been associated with differentiation processes, therefore this could indicate a restriction of lineage commitment mediated by PRMT5^24,25,40,41^.

Besides the expression of growth enhancing pathways, we found PRMT5 to maintain DNA repair pathways, which is in line with reports of PRMT5 affecting DNA repair by influencing the splicing of repair factors^42,43^. Blocking PRMT5 activity in our case, however, resulted in a reduction of BRCA1 levels as well as of additional components of different repair pathways. Thereby, we could prime cells to undergo apoptosis upon additional treatment with the PARP-inhibitor Veliaparib. Inhibitors of Poly(ADP-ribose) polymerases have been used to exploit DNA-repair defects and as therapeutic strategy in BRCA-deficient cancers^44,45^. Sensitizing cells for such inhibitors through the inhibition of PRMT5 might provide additional possibilities of treatment. Also, PRMT5 maintained the expression of the AP1 protein BATF3, which has been described as an upstream MYC-activator in Hodgkin lymphomas and T cell leukemia^26,27^ and is known to cause lymphomas of mature B cells in mice^46^. Our thorough transcriptional analysis identified further molecular interactions with the MYC transcriptional network, supported by additional analysis of clinical data, showing CLL patients with high PRMT5 expression to display enhanced MYC activity. In turn high MYC levels seemed to protect from the effects of PRMT5 inhibition in our system. MYC has long been known as a crucial oncogene in multiple tumor types, including several B-NHLs, like DLBCL or Burkitt lymphoma^47–49^. In CLL few cases of inactivated MYC-antagonists, like MGA, PTPN11 or FUBP1 have been described^6,50^ postulating an important function of the MYC network in this disease. We identified MXD4, an additional antagonist of MYC being repressed by PRMT5, thereby providing a possible explanation for the observed requirement of MYC driven tumors for PRMT5 expression^10^, beyond its role in maintaining MYC and CCND1 expression via activating WNT-signaling^51^. In turn, signaling via AKT/MYC forms a feedforward-loop enhancing PRMT5 function^35^, fitting our observation of high MYC levels apparently having a protective effect on the physiological consequences of PRMT5 inhibition in CLL and MCL cell lines.

PRMT5 gained increasing attention due to its oncogenic functions, while little is known about its initial deregulation during cellular transformation. By reconstructing an event of genomic deregulation for PRMT5 in CLL, we validated our strategy to identify deregulated cancer driving factors in a highly cell type specific manner and contributed to further understanding of PRMT5 function in chronic lymphocytic leukemia.

## Methods

### Cell culture and treatments

All used cell lines were held at 37 °C and 5% CO_2_ unless stated otherwise and authenticated by fingerprinting at the DSMZ (Braunschweig, Germany), cultivated as follows and split every 2–3 days: DAUDI (ACC-78), HG-3 (ACC-765), JEKO-1 (ACC-553), JVM-2 (ACC-12), JVM-13 (ACC-13), MINO (ACC-687), PGA-1 (ACC-766), RAMOS (ACC-603), REC-1 (ACC-584), VAL (ACC-586), WA-C3CD5+ (ACC-769) cells were grown in RPMI 1640 medium with 10% fetal calf serum (FCS) and MEC-1 (ACC-497) in Iscove’s MDM with 10% FCS. 293 T (ACC-635) cells were grown in Dulbecco’s MEM with 10% FCS and at 10% CO_2_. Inhibition of PRMT5 enzymatic activity was achieved with the indicated doses of EPZ015666 (Sigma, SML1421) for 96 h. Additional treatments were performed with the following chemicals: Veliparib (40 µM, 48 h; Selleckchem; S1004), SAHA (1 µM, 48 h; Sigma; SML0061), JQ1 (5 µM, 48 h; Sigma; SML0974), Chaetocin (50 nM, 24 h; Sigma; C9492).

### Assessment of growth, metabolic activity and apoptosis

For the analysis of growth rates 10^4^ cells per well and cell line were seeded in triplicates into 96 well plates and confluence of the whole well was monitored over a total of 96 h every 6 h with the IncuCyte live cell imager (Essen BioScience). Metabolic analyses were performed with a standardized MTT-assay (3-(4, 5-dimethylthiazol- 2-yl)-2, 5-diphenyltetrazolium bromide; Sigma). Cells were grown in the presence of MTT for 4 h and measured in a Spark microplate-reader (Tecan). Induction of apoptosis was monitored with the IncuCyte Caspase-3/7 Red Reagent (Essen BioScience; 4704) for the indicated time and according to manufacturer’s instructions.

### Artificial genomic deletion on chr14

For CRISPR/cas9 mediated genomic engineering, guideRNAs (sequences are listed in Table S3) were designed to each border of the PrHi-C anchor region and cloned into pLentiCRISPR v2^52^ (Addgene; 52961). Lentiviral particles were generated by simultaneous transfection of the gRNA construct with pCMV-VSV-G^53^ (Addgene; 8454) and psPAX2, a kind gift of D. Trono’s lab (Addgene; 12260) into 293 T cells with SuperFect (Qiagen). Target PGA-1 cells were spin-infected on two consecutive days at 800 g for 30 min at 30 °C in the presence of 3 μg/mL polybrene. Selection of transduction positive clones was done by the addition of 0.2 µg/mL puromycin (Sigma). Serial dilutions were performed to isolate individual clones, which were screened for the correct rearrangement with direct PCR (TaKaRa) (primer sequences are listed in Table S3). For detection of the exact breakpoint, PCR-products of positive clones were sequenced on an Applied Biosystems 3500 Genetic Analyzer (Applied Biosystems).

### Protein samples and Western blot analysis

Cell lysates were prepared in RIPA buffer containing protease inhibitors (Sigma) and phosphatase inhibitors (Roche). Protein concentrations were measured with Coomassie Blue Bradford Reagent (ThermoFisher; 23238) and equal amounts of protein were separated on 6–15% polyacrylamide gels and transferred onto PVDF membranes using a mini-Protean system (Biorad). Membranes were blocked in 5% BSA or 3% dry milk powder in TBS + 0.5% Tween-20 and incubated with the following primary antibodies overnight: rabbit polyclonal PRMT5 (Epigentek; A-3005); rabbit polyclonal DAD1 (Novus; IMG-5615); rabbit polyclonal OXA1L (Biozol; LS-C334623); rabbit polyclonal MXD4 (ThermoFisher; PA5-40596); mouse monoclonal c-Myc (ThermoFisher; 13-2500); rabbit polyclonal BRCA1 (Cell Signaling; 9010); mouse monoclonal GAPDH (Abcam; ab8265), mouse monoclonal alpha-tubulin (Sigma; T5168). Washing and staining with either anti-rabbit or anti-mouse secondary antibodies linked to HRP (GE Healthcare; NA934V or NA931V respectively) followed. Detection was done with Clarity Max Western ECL reagent (Biorad) and the digital ChemoStar Imager (INTAS).

Western blot quantifications were performed using the ImageJ software by measuring the mean intensity of each band, performing background subtraction and normalization to the indicated control protein band. Where indicated, the derived values were further divided by the respective control condition. Each experiment was done in independent replicates as indicated and median values are shown together with corresponding SD.

### Expression analysis and RNA-seq

For the analysis of transcript abundance, total RNA was isolated with RNAeasy Kit (Qiagen) and 1 µg of total RNA was subjected to reverse transcription using the Superscript II First strand synthesis kit with random primers (Invitrogen). Semi-quantitative PCR was performed for the indicated genes in a 7500 Fast Real-time PCR System (Applied Biosystems) with the SsoFast EvaGreen Supermix (Biorad). Primers are listed in Supplemental Table S3. RNA-Seq experiments were conducted in two batches: six samples were commissioned for RNA-Seq to GATC Biotech involving TruSeq library preparation and sequencing on Illumina HiSeq2500; six further samples were prepared via SENSE library kit (Lexogen), on Illumina NextSeq500.

### CUT&RUN and CUT&RUN-seq

Cleavage Under Target and Release Using Nuclease (CUT&RUN) was done according to the protocol published by the Henikoff lab with slight modifications^19^: around 200.000 cells per reaction were incubated overnight at 4 °C in antibody-buffer containing 0.05% digitonin (Millipore) and one of the following antibodies: rabbit polyclonal CTCF (Millipore; 07-729), rabbit polyclonal H3K4me3 (Diagenode; C15410003), rabbit polyclonal H3K27ac (Diagenode; C15410196), rabbit polyclonal H3K27me3 (Diagenode; C15410195), after three washsteps, samples were incubated with proteinA-NMase fusion protein (a kind gift of Epicypher) for 1 h at 4 °C. Cleavage around bound target proteins was done at 0 °C for 5 min by addition of calciumchlorid to a final concentration of 10 mM. After removal of the buffer, reaction was stopped by adding 20 mM EGTA in STOP buffer and subsequent release of cut DNA-fragments at 37 °C, followed by phenol-chloroform extraction. For global analysis of binding-sites, 5 ng precipitated DNA bound to each factor of interest was subjected to library preparation with the KAPA Hyperprep Kit (Roche; 07962347001). Indexed adapters were used to pool samples for 75 bp single-end runs on an Illumina NextSeq500. Following sequencing, fastq generation and demultiplexing was done via bcl2fastq (Illumina, v2.17.1.14).

### Data-analysis

Following sequencing and fastq generation, demultiplexing was done via bcl2fastq (v2.17.1.14, Illumina). Reads were trimmed via fastq-mcf (ea-utils 1.04.807) and quality controlled via FastQC (v0.11.5). For gene expression and CUT&RUN-seq analysis reads were aligned by STAR (2.5.3a)^54^ to the Gencode Homo sapiens genome (v26) and converted/sorted via samtools (0.1.19)^55^. For expression analysis the reads were counted to each gene via HTSeq-count python script (0.8.0)^56^. Data was processed and analyzed in the R/Bioconductor environment (3.4.4/3.6). Normalization, estimation of dispersions and testing for differentially expressed genes based on a test assuming negative binomial data distribution was computed via DESeq. 2 (1.18.1)^57^ and enriched by ensembl v89 annotations. Batch effects were considered in the gene expression model and p-values were FDR-corrected. GO annotation and Reactome Pathway analysis were done with the R/bioconductor packages clusterProfiler (3.12.0)^58^ and ReactomePA (1.28.0)^59^, respectively. Additional pathway analysis was done using Ingenuity Pathway Analysis (Qiagen) and GSEA^60^. Prediction of MYC activity using the DoRhotEA algorithm was described previously^22,23^. Graphical representation of genomic loci and data from NGS was generated with ggplot2 (3.2.0) and GVIZ (1.28.0)^61^.

### Pipeline to find genes deregulated by translocations

Data on structural variations from the ICGC CLLE project (v22) was downloaded from the ICGC-web portal (https://dcc.icgc.org/api/v1/download?fn=/release_22/Projects/CLLE-ES/). Regions of genomic aberrations were extracted by extending each chromosomal breakpoint by +/− 50 bp, maintaining respective donor IDs. Genomic coordinates were transferred to hg38 with the UCSC-liftover tool. Similarly, promoter-interactions from PrHi-C were taken from supplementary data from Javierre *et al*.^18^ and genomic coordinates of individual regions were transferred to the same genomic build. Candidate genes were called, if genomic breakpoints located within promoter-interactions of individual genes. For each of such genes, we calculated the DESeq2-normalized median expression and its interquartile range (IQR) across all donors for which data on structural variations was available. Subsequently, we filtered for donors with expression changes in such candidate genes > 2x IQR and with a normalized expression> 10. Additional filtering was done by checking the occurrence of similar disrupted promoter-interactions in at least one additional donor with the corresponding expressional change > 1x IQR. Finally, the derived list of candidates was checked for prognostic effects. Therefore, all donors of the ICGC-CLLE cohort (n = 291), together with the donors described in Herold *et al*.^2^ (n = 107) were grouped according to their expression level of each of the candidate genes and overall survival in the groups of high versus low expressing donors was estimated by Log-rank test, setting a Benjamini-Hochberg corrected padj <5 * 10^−2^ as threshold and visualized as Kaplan-Meier plots (R-package survival, 2.42.6).

### Accession numbers

Sequencing data generated during this study has been deposited at the European Nucleotide Archive and entries can be found via ArrayExpress under E-MTAB-8219 for RNA-seq data and E-MTAB-8203 for CUT&RUN-seq.

## Supplementary information

Supplementary information.

Supplementary information 2.
