# Supplementary material for: Genomic deregulation of PRMT5 supports growth and stress tolerance in chronic lymphocytic leukemia
Source: Sci Rep. 2020 Jun 17;10:9775. doi: 10.1038/s41598-020-66224-1 (PMC7299935; doi:10.1038/s41598-020-66224-1)
Supplement: Supplementary file 1 — Supplementary information. [file 41598_2020_66224_MOESM1_ESM.pdf]

Genomic deregulation of PRMT5 supports growth and stress tolerance in chronic lymphocytic leukemia

Ann-Kathrin Schnormeier[1,2,3], Claudia Pommerenke[1,3], Maren Kaufmann[1], Hans G. Drexler[1] and Max Koeppel[1,*]

[1]Leibniz-Institute DSMZ-German Collection of Microorganisms and Cell Cultures, Department of Human and Animal Cell Lines, Braunschweig, Germany

[2]Present address: Institute for Cell Biology (Tumor Research), University of Duisburg-Essen, Medical School, Germany

[3]These authors contributed equally

[*]corresponding author: Max Koeppel, PhD

Supplemental Material

contains seven supplemental figures and two supplemental tables

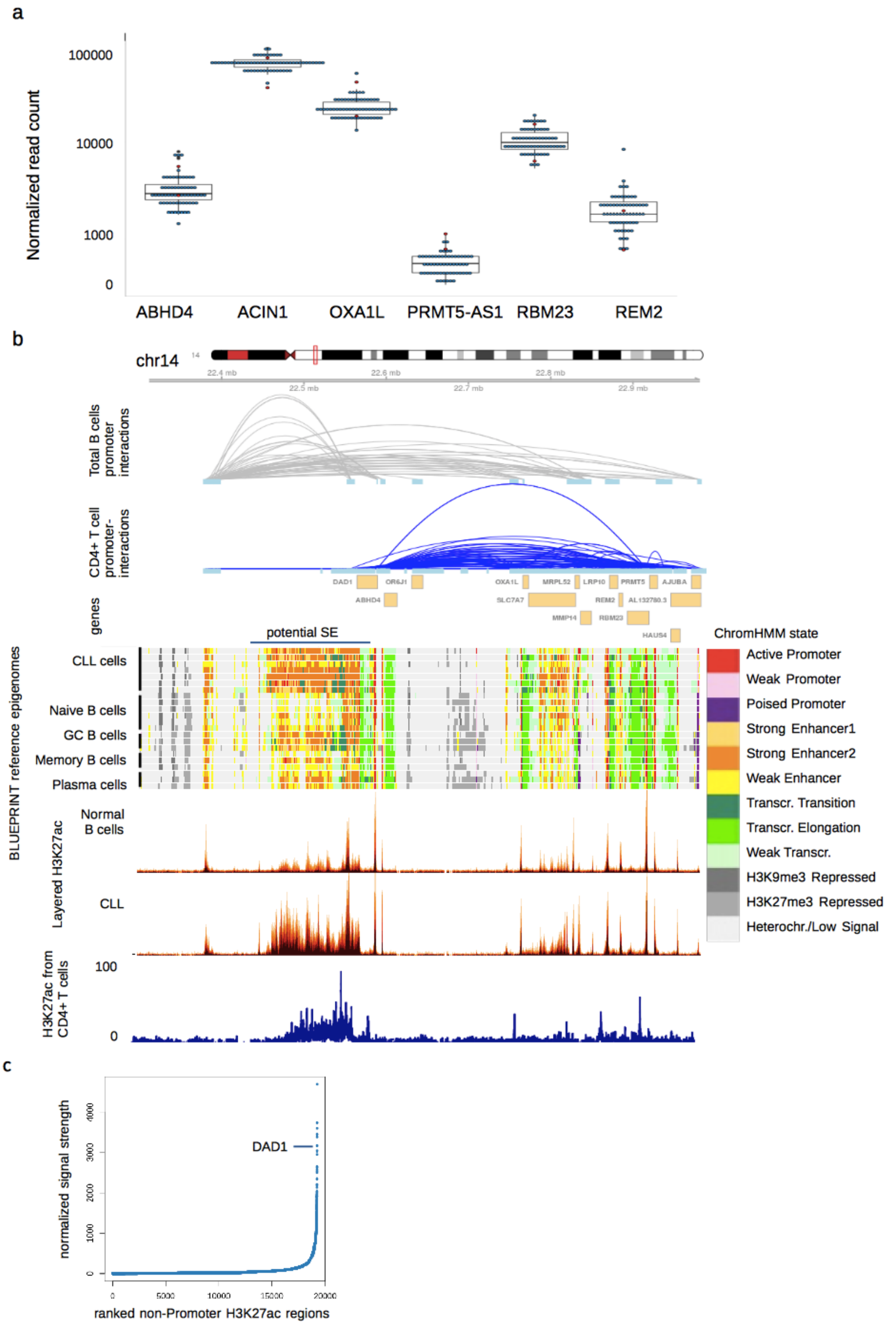

Figure S1 related to **Figure 1: Cell type specific promoter interactions and impact of the loss of the upstream regulatory region on associated genes.** a) Expression level of

additional genes on chr14 interacting with the regulatory region upstream of DAD1 in donors from ICGC-CLLE. Normalized expression is shown for the indicated genes (donors with a breakpoint upstream of DAD1 in red, other donors in blue). b) Schematic representation of the locus on chr14 and its epigenetic make-up. Below the genomic coordinates, promoter-interactions (PrHi-C) from total B cells (grey) and CD4-positive T cells (blue) for the indicated genes are shown (anchor-regions in lightblue, height correspond to published score). Below the gene model chromatin states as defined from BLUEPRINT in Beekman et al.[1] are depicted for the reference set of CLL samples and the indicated B cell subtypes, followed by layered signal of H3K27ac ChIP-seq from B cells and the extended CLL cohort and finally from CD4-positive T cells (darkblue) from BLUEPRINT (from top to bottom). c) Identification of the DAD1 upstream region as potential super enhancer (SE) in HG-3 cells. Regions with enriched H3K27ac from CUT&RUN analysis were ranked by signal-strength after promoter regions were excluded.

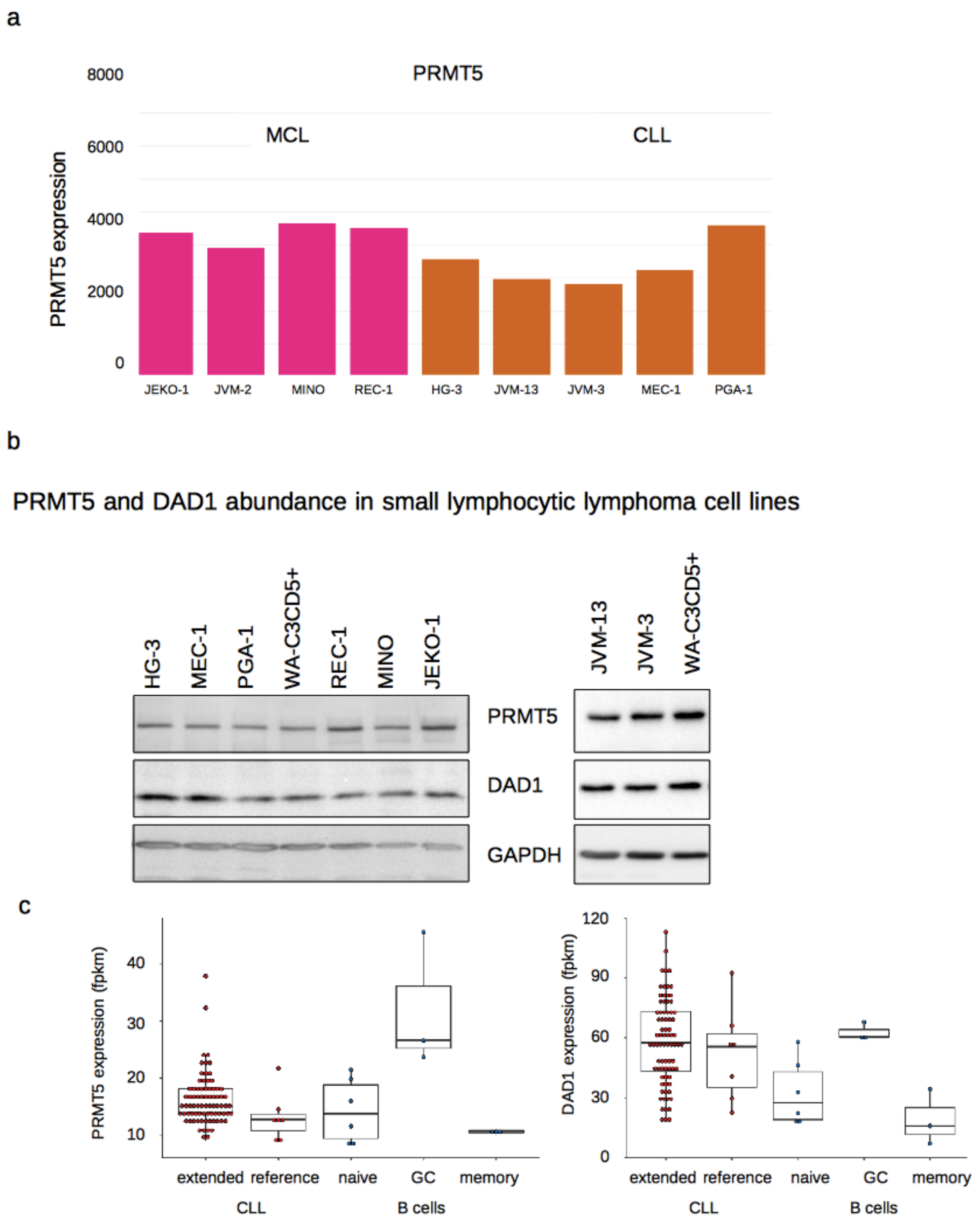

Figure S2 related to **Figure 1: Analysis of PRMT5 in cell lines derived from MCL and CLL.** a) Levels of PRMT5 mRNA as determined by RNA-seq from Quentmeier et al.[2]. b) Whole western blots for PRMT5 and DAD1 in a range of CLL and MCL cell lines. GAPDH served as loading control. c) Expression of PRMT5 (left) and DAD1 (right) from BLUEPRINT (Beekman et al.)[1]. Besides the reference datasets for the indicated B cell subpopulations and the CLL dataset, expression from the extended CLL cohort is also included.

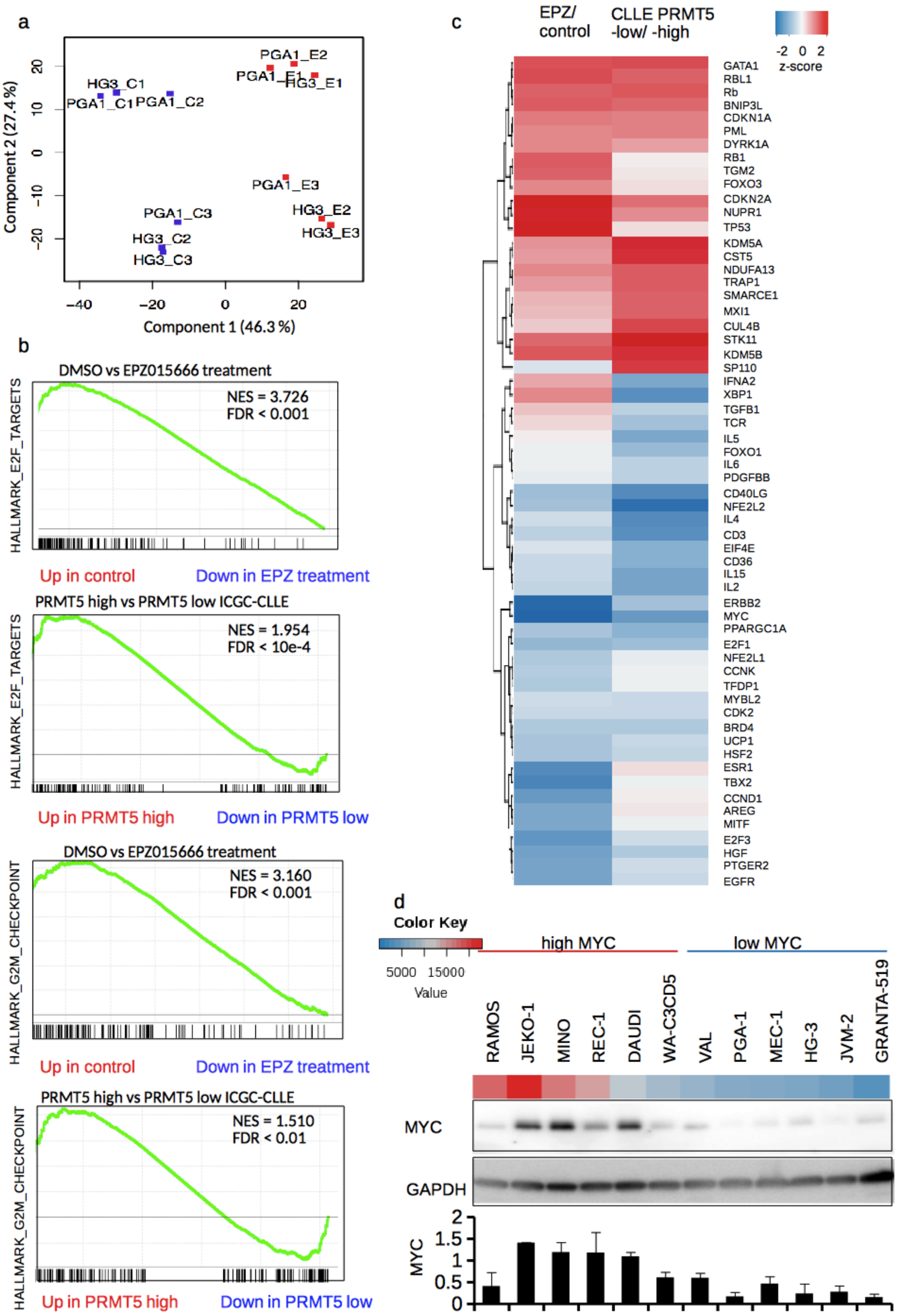

a
Component 2 (27.4 %)
Component 1 (46.3 %)
PGA1_E2
PGA1_E1
HG3_E1
HG3_C1
PGA1_C1
PGA1_C2
PGA1_E3
PGA1_C3
HG3_E2
HG3_E3
HG3_C2
HG3_C3
b
DMSO vs EPZ015666 treatment
HALLMARK_E2F_TARGETS
NES = 3.726
FDR < 0.001
Up in control
Down in EPZ treatment
PRMT5 high vs PRMT5 low ICGC-CLLE
HALLMARK_E2F_TARGETS
NES = 1.954
FDR < 10e-4
Up in PRMT5 high
Down in PRMT5 low
DMSO vs EPZ015666 treatment
HALLMARK_G2M_CHECKPOINT
NES = 3.160
FDR < 0.001
Up in control
Down in EPZ treatment
PRMT5 high vs PRMT5 low ICGC-CLLE
HALLMARK_G2M_CHECKPOINT
NES = 1.510
FDR < 0.01
Up in PRMT5 high
Down in PRMT5 low
c
EPZ/ control
CLLE PRMT5 -low/ -high
-2 0 2
z-score
GATA1
RBL1
Rb
BNIP3L
CDKN1A
PML
DYRK1A
RB1
TGM2
FOXO3
CDKN2A
NUPR1
TP53
KDM5A
CST5
NDUFA13
TRAP1
SMARCE1
MXI1
CUL4B
STK11
KDM5B
SP110
IFNA2
XBP1
TGFB1
TCR
IL5
FOXO1
IL6
PDGFBB
CD40LG
NFE2L2
IL4
CD3
EIF4E
CD36
IL15
IL2
ERBB2
MYC
PPARGC1A
E2F1
NFE2L1
CCNK
TFDP1
MYBL2
CDK2
BRD4
UCP1
HSF2
ESR1
TBX2
CCND1
AREG
MITF
E2F3
HGF
PTGER2
EGFR
d
Color Key
5000 15000
Value
high MYC
low MYC
RAMOS
JEKO-1
MINO
REC-1
DAUDI
WA-C3CD5
VAL
PGA-1
MEC-1
HG-3
JVM-2
GRANTA-519
MYC
GAPDH
MYC

Figure S3 related to **Figure 2: Transcriptional responses upon in vitro PRMT5 inhibition are resembled in vivo.** a) Principal component analysis (PCA) of the RNA-seq samples generated in this study. Indicated are the different cell lines for the control (_c_) and the EPZ015666-treated (_e_) replicates and their respective number. Component 1 reflects the differences from treated to untreated samples, while Component 2 separates samples according to their library preparation method (upper samples: Illumina TrueSeq; lower part: Lexogen SENSE). b) GSEA of genes ranked by expression differences shows a downregulation of the Hallmark Gene Sets E2F_TARGETS and G2M_CHECKPOINT between control and EPZ015666 treated HG-3 and PGA-1 cells as well as between PRMT5 high versus PRMT5 low CLL donors. c) IPA upstream regulator analysis indicates transcriptional regulators with changed activity upon treatment of cells with EPZ015666 or in PRMT5 high versus PRMT5 low CLLE donors (pval < 0.001 and zscore $> |2|$). d) Level of MYC mRNA was used to rank indicated cell lines of CLL and MCL origin as well as Burkitt lymphoma cell lines, determined by RNA-seq. Western blot depicts protein levels of MYC, GAPDH served as loading control, quantifications were performed using ImageJ software and signal was normalized to GAPDH (Error Bars depict SD from two biological replicates).

a

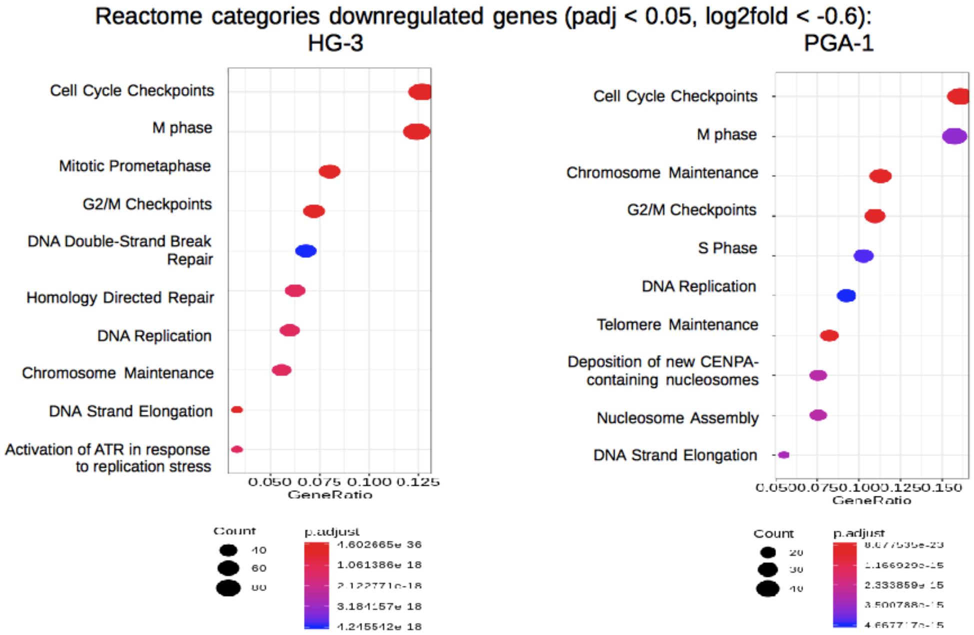

b

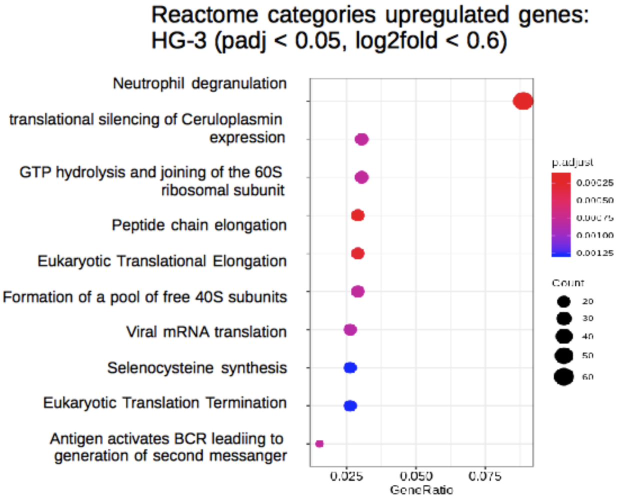

Figure S4 related to **Figure 3: Reactome Analysis for up- and down-regulated genes indicated different affected pathways.** Reactome Pathway annotation of genes changing in expression in PGA-1 and HG-3 cells upon PRMT5 inhibition. Cells were treated for 96 h with 10 μM of PRMT5 inhibitor prior to RNA-seq analysis. a) Downregulated genes as defined by DESeq2 (padj < 0.05, logFC < -0.6) in either HG-3 (left) or PGA-1 (right) cells. Top10 categories are shown. b) Upregulated genes as defined by DESeq2 (padj < 0.05, logFC > 0.6) in HG-3 cells. Top10 categories are shown.

a

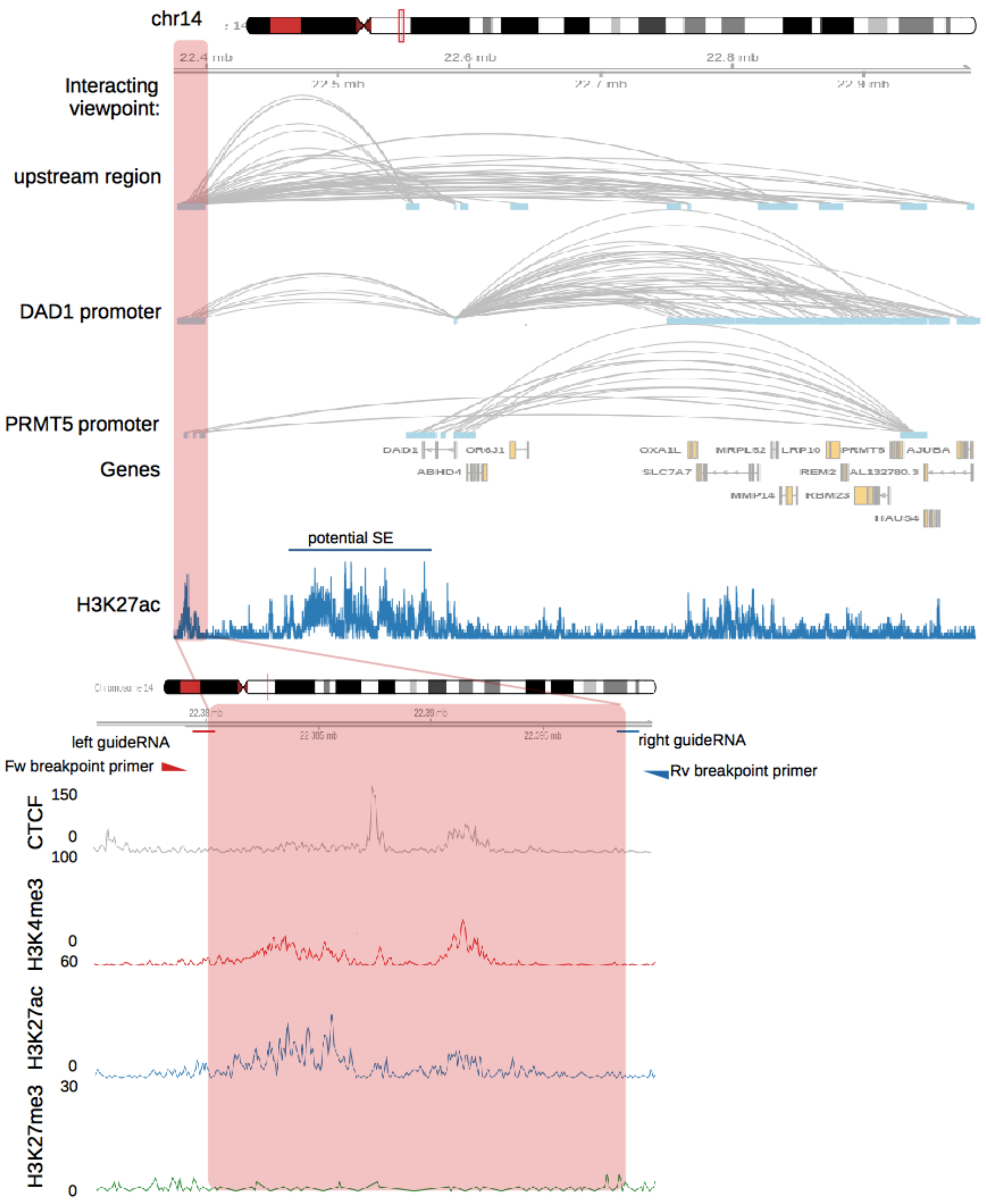
chr14
22.4 mb
22.6 mb
22.8 mb
Interacting viewpoint:
upstream region
DAD1 promoter
PRMT5 promoter
Genes
DAD1
OR6J1
ABHD4
OXA1L
MRPL52
LRP10
PRMT5
AJUBA
SLC7A7
REM2
MMP14
potential SE
H3K27ac
left guideRNA
right guideRNA
Fw breakpoint primer
Rv breakpoint primer
CTCF
150
0
H3K4me3
100
0
H3K27ac
60
0
H3K27me3
30
0

b

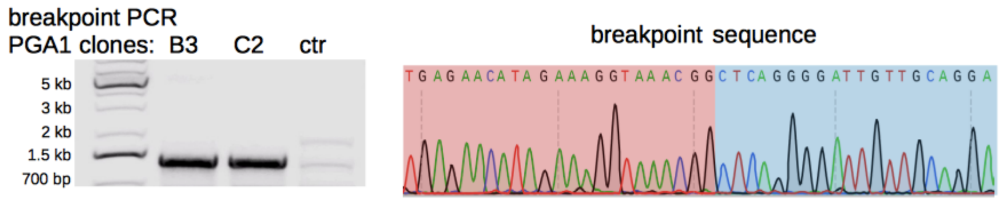
breakpoint PCR
PGA1 clones: B3 C2 ctr
5 kb
3 kb
2 kb
1.5 kb
700 bp
breakpoint sequence
TGAGAACATAGAAAGGTAAACGGCTCAGGGGATTGTTGCAGGA

Figure S5 related to **Figure 4: Deletion of the upstream regulatory region.** a) Interactions between different regions in the locus on chr14 as identified by Promoter-Capture Hi-C. From top to bottom indicated are the interactions of the upstream region with gene promoters in the downstream locus, interactions of the DAD1 promoter and the PRMT5 promoter. Light red box highlights the upstream regulatory region and the epigenetic profile generated by CUT&RUN in HG-3 cells. Also shown are the locations of the CRISPR/cas9 guideRNAs and the primers used for breakpoint PCR (lower panel). b) Results of the breakpoint PCR in two deletion clones and the non-guideRNA control (left) and a result from sequencing the breakpoint PCR product (right).

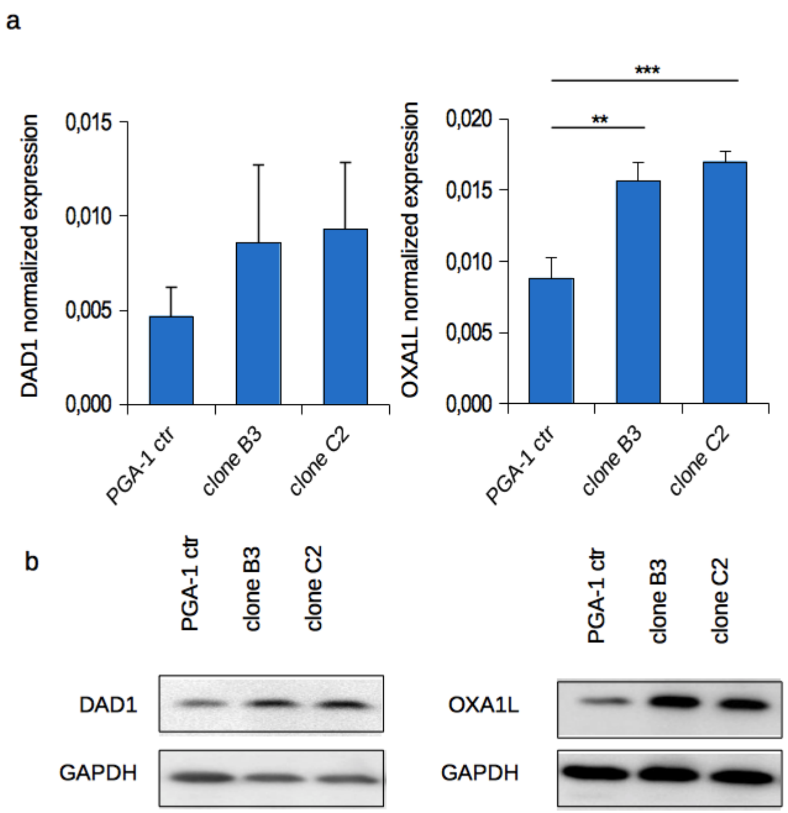

DAD1 over PGA-1 ctr.	1.81	1.54
SD	0.50	0.45

OXA1L over PGA-1 ctr.	1.37	1.52
SD	0.31	0.25

Figure S6 related to **Figure 4: Expressional changes in DAD1 and OXA1L upon loss of upstream regulatory region.** a) Normalized expression levels of the indicated genes in PGA-1 control cells and in PGA-1 clones with a CRISPR/cas9 engineered deletion of the upstream regulatory region on chr14; error bars derived from three biological replicates; statistical differences were analyzed by student's paired T-test (** $p < 0.005$; *** $p < 0.001$). b) Western blot for DAD1 and OXA1L in the same PGA-1 clones lacking the upstream regulatory region. GAPDH served as loading control, quantifications for the indicated proteins below the blots were done with ImageJ software, normalized to loading control and calculated over the non-targeted control clone (SD from three biological replicates is also listed).

Full length blots

Fig 1e

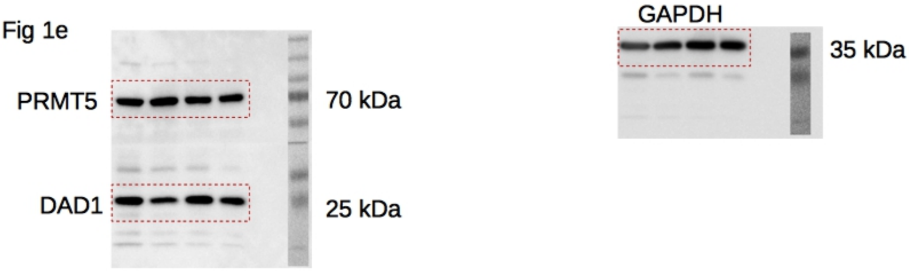

Fig 2d

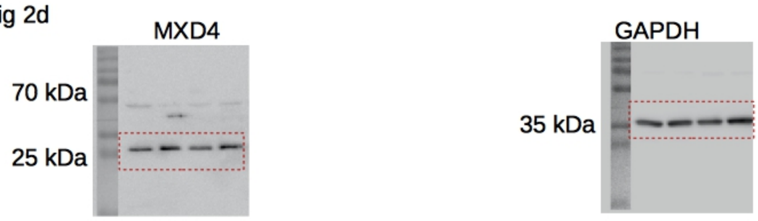

Fig 3c

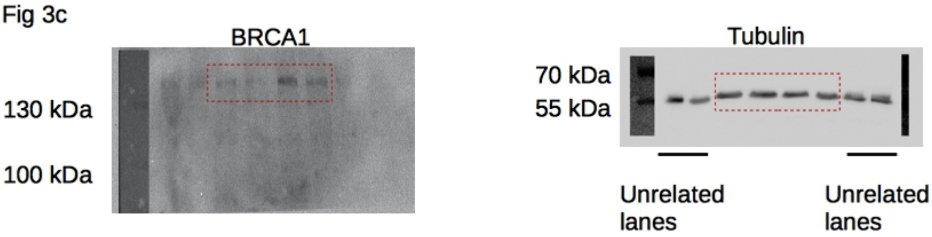

Fig 4c

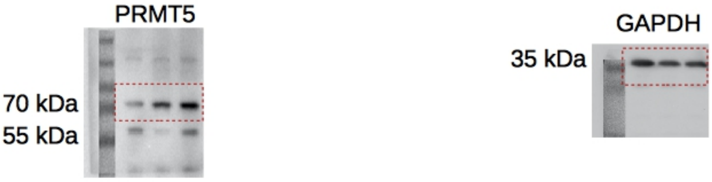

Full length blots (continued)

Fig S2b

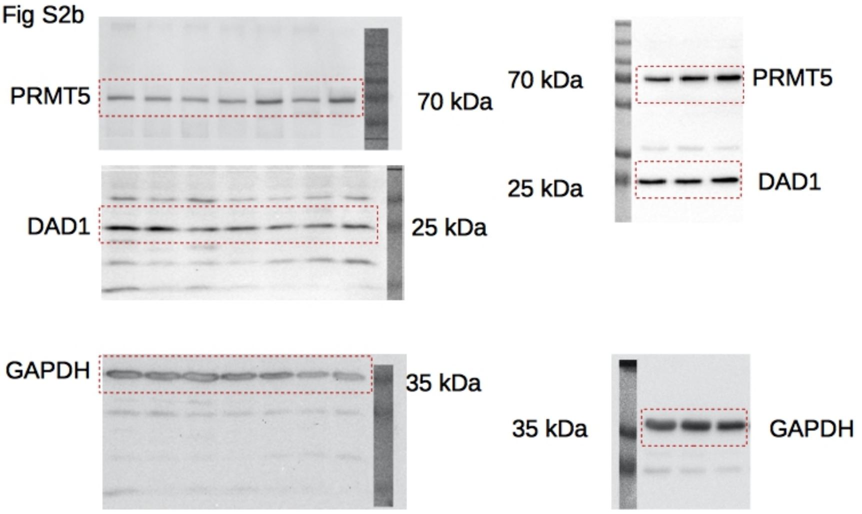

Fig S3d

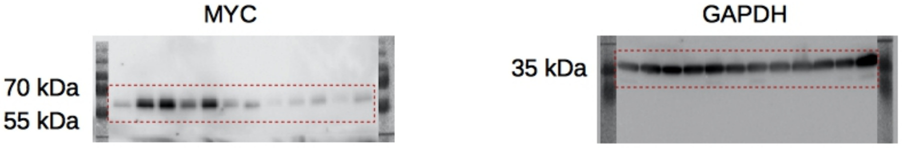

Fig S6b

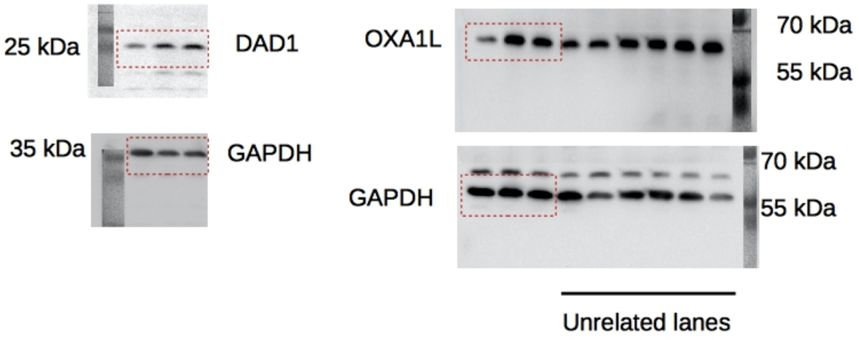

Supplemental Fig. S7, related to **Fig. 1e, Fig. 2d, Fig. 3c, Fig. 4c, Fig. S2b, Fig. S3d** and **Fig. S6b:** Full size blots of protein analysis shown in the respective figures.

Membranes have either stained consecutively for the different proteins, or where applicable cut between the expected protein sizes. Red dashed boxes indicate bands used in the figures.

Table S1

Clinical characteristics of the PRMT5 high and PRMT5 low expressing ICGC-CLLE donors

**ICGC-CLLE**	**PRMT5-high group (n = 146)**	**PRMT5-low group (n = 145)**
IGHV_UNMUT	65	40
IGHV_MUT	81	105
tri12	16	28
del13q14	91	91
del11q22-23	26	15
del17p13	9	6
mutNOTCH1	12	23
mutTP53	12	1
mutATM	16	8
mean OS [month]	80	78

Table S3

Primers used in this study.

guideRNAs for CRISPR/cas9 deletion of PCHi-C anchor region	
chr14-left_1_f2	CACCGAAGGTAAACGGCTCGAGAGA
chr14-left_1_r2	AAACTCTCTCGAGCCGTTTACCTTC
chr14-right_2_f2	CACCGATTACAATAACACCTACCTC
chr14-right_2_r2	AAACGAGGTAGGTGTTATTGTAATC
breakpoint PCR and breakpoint sequencing primers	
chr14-left_BP_fw	TTTTCTCCCCCTGACACTTG
chr14-left_BP_rv	TCCATCAGGCTAGTGTTGTCC
chr14-right_BP_fw	TGAGCCATTTACTGCTGTGC
chr14-right_BP_rv	GAGGCTGAAGTGGAGGTGTC
chr14-left_BP_fw	TTTTCTCCCCCTGACACTTG
primers for RT-qPCR	
GAPDH_fw	GAAGGTGAAGGTCGGAGTC
GAPDH_rv	GAAGATGGTGATGGGATTTC
ACTB_ex_f	ACCCAGCACAATGAAGATCA
ACTB_ex_r	TCGTCATACTCCTGCTTGCT
PRMT5_fw	TTGGAATTTCCTGTGGAGGT
PRMT5_rv	AGAGGATGGGAAACCATGAG
DAD1_fw	TGCACCTTGTTGTCATGAACT
DAD1_rv	CAATAAGCTGCCATCTCCAG
CCL22_ex_f	CGCGTGGTGAAACACTTCTA
CCL22_ex_r	GCTCTTCATTGGCTCAGCTT
LIMA1_ex_f	CTGCTTCCGTTGCTCCTATT
LIMA1_ex_r	GCCCAAAGCCTTCATCATAG
KLHL24_ex_f	TGGTGGAGGACCTGATGATA
KLHL24_ex_r	CCTTTTGGCAATTGGGATAG
PIK3IP1_ex_f	CGTTCAATCACCTGCAAGAC
PIK3IP1_ex_r	GGTACAGGTGGCCGTTGT
BATF3_ex_f	gaagaagcagacccagaagg
BATF3_ex_r	aggtgcttcagctcctctgt
KLF2_ex_f	TCACACCTGCAGCTACGC
KLF2_ex_r	GTTGCAGTGGTAGGGCTTCT
FCRL5_ex_f	TCATATCTGACAGCCCGAGA
FCRL5_ex_r	AAGTGCGCAGAGAATCTTCC
LRRC20_ex_f	GGAACCAGTTCCAGGACTTC
LRRC20_ex_r	GTTGAAGCGGAGGTTGATG

1 Beekman, R. *et al*. The reference epigenome and regulatory chromatin landscape of chronic lymphocytic leukemia. *Nat Med* **24**, 868-880, doi:10.1038/s41591-018-0028-4 (2018).

2 Quentmeier, H. *et al*. The LL-100 panel: 100 cell lines for blood cancer studies. *Sci Rep* **9**, 8218, doi:10.1038/s41598-019-44491-x (2019).
